# Universal Adaptive Neural Network Predictive Algorithm for Remotely Piloted Unmanned Combat Aerial Vehicle in Wireless Sensor Network

**DOI:** 10.3390/s20082213

**Published:** 2020-04-14

**Authors:** Hongyang Xu, Guicai Fang, Yonghua Fan, Bin Xu, Jie Yan

**Affiliations:** 1 School of Astronautics, Northwestern Polytechnical University, Xi’an 710072, China; xhyang@mail.nwpu.edu.cn (H.X.); jyan@nwpu.edu.cn (J.Y.); 2 China Aerodynamics Research and Development Center, Mianyang 621000, China; breezechar@163.com; 3 School of Automation, Northwestern Polytechnical University, Xi’an 710072, China; binxu@nwpu.edu.cn

**Keywords:** unmanned combat aerial vehicle (UCAV), wireless sensor network (WSN), time delay, neural network observer, state predictor

## Abstract

Remotely piloted unmanned combat aerial vehicle (UCAV) will be a prospective mode of air fight in the future, which can remove the physical restraint of the pilot, maximize the performance of the fighter and effectively reduce casualties. However, it has two difficulties in this mode: (1) There is greater time delay in the network of pilot-wireless sensor-UCAV, which can degrade the piloting performance. (2) Designing of a universal predictive method is very important to pilot different UCAVs remotely, even if the model of the control augmentation system of the UCAV is totally unknown. Considering these two issues, this paper proposes a novel universal modeling method, and establishes a universal nonlinear uncertain model which uses the pilot’s remotely piloted command as input and the states of the UCAV with a control augmentation system as output. To deal with the nonlinear uncertainty of the model, a neural network observer is proposed to identify the nonlinear dynamics model online. Meanwhile, to guarantee the stability of the overall observer system, an adaptive law is designed to adjust the neural network weights. To solve the greater transmission time delay existing in the pilot-wireless sensor-UCAV closed-loop system, a time-varying delay state predictor is designed based on the identified nonlinear dynamics model to predict the time delay states. Moreover, the overall observer-predictor system is proved to be uniformly ultimately bounded (UUB). Finally, two simulations verify the effectiveness and universality of the proposed method. The results indicate that the proposed method has desirable performance of accurately compensating the time delay and has universality of remotely piloting two different UCAVs.

## 1. Introduction

At present manned/unmanned formation is the developing trend of future war, the unmanned combat aerial vehicle (UCAV) can remove the physical restraint of the pilot, maximize the performance of the air fighter and effectively reduce casualties. However, restricted by the development of current intelligence technology, the UCAV still cannot complete the tasks independently. Therefore, a remotely piloted UCAV will be a prospective mode of air fight in the future, and remotely piloted technology has become an important way to win unmanned aerial combat [1,2,3].

**Figure 1:**
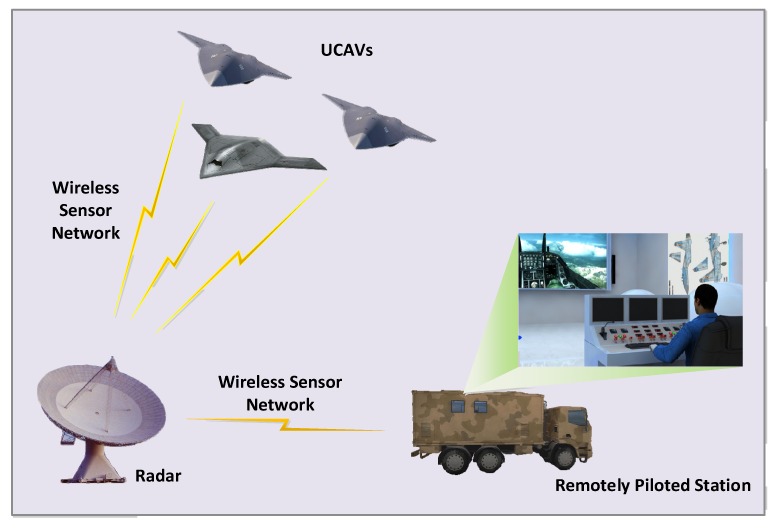
The structure of the wireless combat sensor network scenario.

The remotely piloted system consists of UCAVs, the wireless sensor network (WSN) and the remotely piloted station (seen in Figure 1). The procedure is as follows: the pilot in the remotely piloted station gives the real-time command according to the combat situations displayed on the interface through the downstream channel of the WSN. Then the remotely piloted command is sent to the UCAV through the upstream channel of the WSN. The UCAV control augmentation system tracks the remotely piloted command rapidly and changes the states of the UCAV [4,5]. However, it has two difficulties in this mode: (1) There is greater time delay in the network of a pilot-wireless sensor-UCAV, which can cause pilot-induced oscillation and engender the phenomenon of “chasing-swing”, and can even cause instability of the loop of remote pilot and UCAV. (2) Each UCAV has its own control augmentation system, and different UCAVs have different aerodynamic and control augmentation systems, including controller parameters and feedback states [6]. Thereby it is very important that the design of a universal state predictive method realizes remotely piloting different UCAVs, even if the parameters of the control augmentation systems are totally unknown.

For the problem of time delay, many researchers have studied and proposed different methods. The predictive approach is very efficient for calculating the time delay [7,8]. To predict the state parameter precisely, different predictive methods have been investigated, such as the Smith predictive method [9,10,11], a Kalman estimator [12,13], feed-forward control [14], reference model based control [15,16], and a state space observer approach [17,18]. Thurling and Greene [19] gave a detailed description of the composition of the delay of a low speed unmanned aerial vehicle (UAV) remotely piloted system and analyzed the result of time delay impacting on the handling qualities; they described how the greater time delay could cause the pilot to over-operate the UAV and even lead to UAV instability. Based on the deterministic linear model and the deterministic delay, the delayed UAV flight state is predicted by solving the state equation. However, this method is too ideal to improve the UAV flight quality in the presence of disturbance and time-varying delay. Teng and Grant [20] improved this algorithm and, based on a low speed UAV linear longitudinal model, adapted the predictor model for real-time using online parameter estimation techniques; then they designed a constant time delay Smith predictor. However, the time delay is always a time-varying system in the WSN. Recently, there are also some researches on time-varying delay prediction. In [21], a kind of state observer based on a deterministic model was presented for a velocity-sensorless vertical take-off and landing aircraft with bounded time-varying delay in its measurement outputs. The proposed observer effectively improved the output measurement delay and the asymptotic convergence property of the estimation error based on the Lyapunov–Razumikhin theorem was proved. In [22], a fuzzy observer-based hybrid force/position predictive method was investigated for a bimanual robot teleoperation system in the presence of dynamics uncertainties and random network-induced time delays. Lu et al. considered the dynamics uncertainties of a slave and the random time delays, therefore, the method is more applicable. There have been many researches on time delay prediction, including deterministic objects, objects with parameter uncertainties, fixed delay time and random delay time systems. However, all of these studies [19,20,21,22] can be classified as networked control problems of low dynamic objects. That is to say, the operator sends the control command directly to the remote actuator of the passive object through the WSN. For networked control systems, only the dynamics model of the controlled object itself is needed, which is relatively simple and usually known [23,24,25].

Due to the need of strong maneuverability, the general aerodynamics of the UCAV is unstable; the UCAV requires its own control augmentation system to guarantee self-stability [26,27]. Consequently, the response of the UCAV is highly dynamic. The remotely piloted mode is different from common remote manipulation such as performed by a manipulator of a satellite or moon rover, which can work on the mode of “do and wait”, and the manipulating commands can be sent to the actuator directly. In remotely piloted mode, for the pilot, the control object is no longer the UCAV itself, but the whole UCAV with the control augmentation system [7]. Therefore, the dynamics model of the UCAV will contain not only the aerodynamic and motion characteristics, but also the control augmentation system and the nonlinear characteristics of the actuator. In addition, in the whole flight envelope, the characteristics of the control augmentation system change with the change of flight conditions and mission; also the control augmentation systems designed by different designers are very different [28]. These problems make it very difficult to describe the dynamics model of the UCAV accurately [29]; this brings much bigger challenges to remotely pilot different UCAVs.

For this problem, many scholars have focused on the model uncertainty and nonlinear unmodeled dynamics. The intelligent algorithm using a fuzzy logic system and a neural network as an approximate estimator is widely studied [30,31,32]. For intelligent algorithms, some recent works can be found in [33,34,35]. A robust adaptive fuzzy tracking controller for a hypersonic flight vehicle subject to both parametric uncertainties and unmodeled dynamics is proposed in [36]. Considering parameter uncertainties and unmodeled dynamics, a full state tracking error system is built. A fuzzy logical system is utilized to identify the nonlinear system with parameter uncertainty and unmodeled dynamics. In [37], a fault-tolerant controller of a hypersonic flight vehicle is presented using back-stepping and composite learning. To obtain good uncertainty approximation, composite learning is proposed for the update of neural weights by constructing a serial-parallel estimation model. The intelligent algorithm is applied to a hypersonic vehicle [38], an autonomous underwater vehicle [39], a UAV [40], a quad rotor [41], a re-entry vehicle [42], etc. Although there are many intelligent algorithm studies on system uncertainties, to our best knowledge, there are few studies related to remotely piloted techniques for a UCAV with a control augmentation system.

Compared with previous studies on remotely piloted UCAVs, this paper is an extension of the previous method proposed in [7]. A predictive method is presented based on the assumption of a linear approximation model in [7]. The method in this paper can solve the state delay prediction problem of the UCAV nonlinear uncertain model, which is more universal. The main contributions of this paper are listed as follows.

(1) A novel universal input–output nonlinear dynamics model of a UCAV with a control augmentation system is established. Considering that the pilot often pays attention to the position information when piloting a UCAV, and the position is just twice the integral of acceleration, which is universal for any UCAV. Consequently, we establish a universal nonlinear uncertain model which uses the pilot’s remotely piloted command as input and the states of the UCAV with a control augmentation system as output. In addition, the nonlinear characteristics of the UCAV’s actuator are also considered.
(2) To deal with the nonlinear unmodeled dynamics, a radial-basis-function (RBF) neural network method is applied to identify the nonlinear dynamics model online. Meanwhile, to guarantee the stability of the overall observer system, an adaptive law is designed to tune the neural network weights.
(3) A time-varying state predictor is designed based on the identified nonlinear dynamics model to achieve accurate predictions of the time delay states.
(4) The overall adaptive neural network observer-predictor scheme is shown to be uniformly ultimately bounded (UUB) via the Lyapunov approach. The predicted errors are proved to converge by analyzing the trajectory. Two simulation results show that the design predictor can identify the nonlinear dynamics model precisely and can predict the flight states effectively.

The remainder of this paper is organized as follows. Section 2 describes the nonlinear dynamics model of the UCAV with a control augmentation system and presents several basic assumptions. In Section 3, a state predictor based on adaptive neural network dynamics is presented, and then the closed-loop system stability is proved. The simulation results and some discussions are presented in Section 4. Finally, conclusions are given in Section 5.

## 2. Problem Formulation

### 2.1. Dynamics Model of a UCAV

In this paper, we use the nonlinear longitudinal dynamics model of a UCAV built in [7]; the details are shown as follows:

(1) $$\begin{array}{l} a_{y}=\frac{\rho V^{2}\bar{S}C_{L}\left(\alpha ,\delta_{e},Ma\right)}{2m}-g\cos\left(\vartheta -\alpha \right)\\ \dot{\alpha }=-\frac{1}{2mV}\rho V^{2}\bar{S}C_{L}\left(\alpha ,\delta_{e},Ma\right)+q+\frac{g}{V}\cos\left(\vartheta -\alpha \right)\\ \dot{q}=\frac{\rho V^{2}\bar{S}\bar{c}C_{M_{yy}}\left(\alpha ,\delta_{e},Ma\right)}{2I_{yy}}\\ \dot{\vartheta }=q\\ {\dot{V}}_{y}=a_{y}\\ \dot{H}=V_{y} \end{array}$$

where H, V, Ma represent altitude, velocity and Mach, respectively, α, ϑ and q are angle of attack, pitch angle and pitch rate, $V_{y}$ and $a_{y}$ denote the longitudinal velocity and acceleration, $I_{yy}$ and m stand for the moment of inertia and the vehicle mass, g is the acceleration owing to gravity, ρ denotes air density, $\bar{S}$ and $\bar{c}$ denote reference area and reference length and $\delta_{e}$ is elevator deflection, which is generated by the UCAV control augmentation system. $C_{L}\left(\alpha ,\delta_{e},Ma\right)$ and $C_{M_{yy}}\left(\alpha ,\delta_{e},Ma\right)$ denote lift coefficient and pitch moment coefficient, respectively; they are both nonlinear functions of angle of attack, elevator deflection and Mach. See Appendix A for a list of symbols.

### 2.2. Dynamics Transformation

In the previous section, the longitudinal dynamics model of UCAV is given. However, the strong maneuverability of the UCAV is usually unstable, and it has its own control augmentation system. Therefore, the object piloted is not the UCAV dynamics itself, but the whole UCAV after the control augmentation. Different from the remote piloted low-speed UAV, the remote piloted command sent by the pilot is no longer the elevator deflection, but the longitudinal acceleration command.

**Figure 2:**
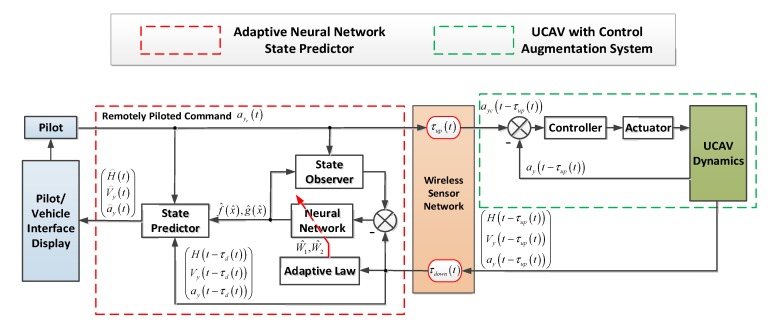
The structure of the pilot-wireless sensor-UCAV closed-loop system, where UCAV is an unmanned combat aerial vehicle.

Figure 2 shows the structure of the pilot-wireless sensor-UCAV closed-loop system. It can be seen that the UCAV control augmentation system consists of two parts: controller and actuator. The controller of the UCAV calculates the elevator deflection command according to the control algorithm designed by the designer to track the remotely piloted command $a_{yc}$. Then, the actuator deflects the corresponding elevator deflection angle to make the UCAV movement. However, because the response characteristic of the UCAV’s controller is greatly variable with flight altitude and velocity, and the backlash and dead zone of the UCAV’s actuator are nonlinear, it is very difficult to accurately describe the dynamics model of the UCAV with a control augmentation system.

Therefore, in this paper, the dynamics model, from the pilot’s acceleration command to the acceleration response of the UCAV, is modeled as a nonlinear uncertain model, and it will be approximated in Section 3. Moreover, due to the pilot often paying attention to the position information when piloting the UCAV, and the position being just twice the integral of acceleration, which is universal for any UCAV, then we can establish a universal nonlinear uncertain dynamics model as follows:

(2) $$\begin{array}{l} \dot{x}\left(t\right)=Ax\left(t\right)+b\left[\chi \left(x\left(t\right),u\left(t\right),t\right)+d\left(t\right)\right]\\ y\left(t\right)=C^{T}x\left(t\right) \end{array}$$

where $x\left(t\right)={\left[\begin{array}{ccc} x_{1}\left(t\right) & x_{2}\left(t\right)\; & x_{3}\left(t\right) \end{array}\right]}^{T}={\left[\begin{array}{ccc} H & V_{y} & a_{y} \end{array}\right]}^{T}\in R^{3}$, $u\left(t\right)=a_{yc}\left(t\right)\in R$ and $y\left(t\right)=x\left(t\right)\in R^{3}$ are state vector, input and output vector, respectively. The unknown disturbance is d(t), with a known upper bound $b_{d}$. The coefficient matrixes of the system are

$$A=\left[\begin{array}{ccc} 0 & 1 & 0\\ 0 & 0 & 1\\ 0 & 0 & 0 \end{array}\right],\;b={\left[\begin{array}{ccc} 0 & 0 & 1 \end{array}\right]}^{T},\;C=\left[\begin{array}{ccc} 1 & 0 & 0\\ 0 & 1 & 0\\ 0 & 0 & 1 \end{array}\right].$$

**Remark** **1.** *In Equation (2),* χ(x(t),u(t),t) *denotes the dynamics model from pilot’s longitudinal acceleration command* $a_{yc}$ *to the response of the UCAV’s longitudinal acceleration* $a_{y}$ *via the control augmentation system. The dynamic process is not only related to the aerodynamic and motion characteristics of the UCAV itself, but also to the response performance of the control augmentation system and the nonlinear characteristics of the actuator. In addition, due to the strong maneuverability of UCAV, its aerodynamic characteristics are very complex, and the couplings between aerodynamic parameters and flight dynamics are strong, thus* χ(x(t),u(t),t) *is an unmodeled dynamics in this paper, and it will be approximated later.*

The latency in the pilot-wireless sensor-UCAV closed-loop system is defined as the time elapsed from pilot’s command until an expected state feedback is displayed in the pilot/vehicle interface [7]. The time delay of the whole closed-loop system primarily includes two parts: uplink time delay $\tau_{up}\left(t\right)$ and downlink time delay $\tau_{down}\left(t\right)$, which vary with the flight distance.

**Remark** **2** **[23].** *According to [23], the total time delay is equal to the delay displayed by the pilot/vehicle interface, therefore, $\tau_{up}\left(t\right)$ and* $\tau_{down}\left(t\right)$ *can be combined as* $\tau_{d}\left(t\right)$ *to make it easier for analysis. Then, the time delay model can be simplified as follows:*

(3) $$\tau_{d}\left(t\right)=\tau_{up}\left(t\right)+\tau_{down}\left(t\right).$$

**Assumption** **1** **[23].** *For the time-varying delay $\tau_{d}\left(t\right)$, we assume that the delays are bounded, known and identical; for different outputs throughout the whole paper, the delay should be bounded by:*

$$\tau_{d}\left(t\right)\in \left[0,\Delta \right].$$

To achieve a convenient expression in the following section, the dynamics model of a UCAV with time-varying delay can be expressed as

(4) $$\begin{array}{l} \dot{x}\left(t\right)=Ax\left(t\right)+b\left[\chi \left(x\left(t\right),u\left(t\right),t\right)+d\left(t\right)\right]\\ \bar{y}\left(t\right)=C^{T}\bar{x}\left(t\right)=C^{T}x\left(t-\tau_{d}\left(t\right)\right) \end{array}$$

where $\bar{y}\left(t\right)={\left[\begin{array}{ccc} \bar{H}\left(t\right) & {\bar{V}}_{y}\left(t\right) & {\bar{a}}_{y}\left(t\right) \end{array}\right]}^{T}=y\left(t-\tau_{d}\left(t\right)\right)$ is the output with measurement time-varying delay.

### 2.3. Preliminaries

In this paper, the unknown functions will be approximated by an RBF neural network. It has been proven that for an arbitrary real continuous nonlinear function λ(ξ) on a compact set $\Omega_{\xi }\subset R^{n}$, there exists the following RBF neural network and ideal weights vector $W^{*T}$, such that:

(5) $$\lambda \left(\xi \right)=W^{*}{}^{T}\Phi \left(\xi \right)+\varepsilon \left(\xi \right),$$

where the input vector is $\xi ={\left[\xi_{i}\right]}^{T}$, i=1,2,…,n is the neural network input dimension, and the neural network approximation error ε(ξ) is bounded by the known constant $\varepsilon_{N}$. Function Φ is the Gaussian function, which is in the following form:

(6) $$\Phi_{j}\left(\xi \right)=\exp\left(-\frac{{\left\|\xi -\eta_{j}\right\|}^{2}}{2\sigma_{j}^{2}}\right),$$

where $\eta =\left[\eta_{ij}\right]=\left[\begin{array}{ccc} \eta_{11} & \cdots & \eta_{1m}\\ \vdots & \ddots & \vdots \\ \eta_{n1} & \cdots & \eta_{nm} \end{array}\right]$ and $\sigma_{j}$, j=1,2,…,m are the center vector and the width of the Gaussian function, respectively.

Then the unknown function λ can be approximated by an RBF neural network. For the network input ξ, the output is given by

(7) $$\hat{\lambda }\left(\xi \right)={\hat{W}}^{T}\Phi \left(\xi \right),$$

where $\hat{W}$ is the estimation of the ideal neural network weight that is provided by some weight-tuning algorithms.

**Remark** **3** **[43].***In this paper, we denote*‖·‖*as the Euclidean norm, and*${\left\|\cdot \right\|}_{F}$ as the Frobenius norm.

**Definition** **1** **[43].***We say that the solution of a dynamic system is UUB if for a compact set*U*of*$R^{n}$*and for all*$x\left(t_{0}\right)=x_{0}\in U$*there exists a*μ>0*and a number*$T\left(\mu ,x_{0}\right)$*such that*‖x(t)‖<μ*for all*$t\geq t_{0}+T$.

## 3. Predictor Design

The greater transmission time delay existing in the pilot-wireless sensor-UCAV closed-loop system causes pilot-induced oscillation and degrades the pilot/vehicle system stability and tracking performance. Therefore, a time-varying state predictor is designed in this section to predict the time delay states. However, because the dynamics model of the UCAV’s control augmentation system is unknown, an adaptive RBF neural network observer is proposed to approximate the unknown nonlinear function. Moreover, a corresponding lemma and the convergence proof of predicted errors are presented, respectively.

### 3.1. State Predictor

The proposed predictor performs according to the following structure:

(8) $$\begin{array}{l} \dot{\overset{⌒}{x}}=A\overset{⌒}{x}+b\left[\chi \left(\overset{⌒}{x}\left(t\right),u\left(t\right),t\right)\right]+K\left(\bar{y}-C^{T}\overset{⌒}{x}\left(t-\tau_{d}\left(t\right)\right)\right)\\ \bar{\overset{⌒}{y}}=C^{T}\overset{⌒}{x}\left(t-\tau_{d}\left(t\right)\right) \end{array}$$

For the three-order systems Equation (4), the predictor is specifically expressed as:

(9) $$\{\begin{array}{l} \dot{\overset{⌒}{H}}\left(t\right)={\overset{⌒}{V}}_{y}\left(t\right)+K_{1}\left(\bar{H}-\overset{⌒}{H}\left(t-\tau_{d}\left(t\right)\right)\right)\\ {\dot{\overset{⌒}{V}}}_{y}\left(t\right)={\overset{⌒}{a}}_{y}\left(t\right)+K_{2}\left({\bar{V}}_{y}-{\overset{⌒}{V}}_{y}\left(t-\tau_{d}\left(t\right)\right)\right)\\ {\dot{\overset{⌒}{a}}}_{y}\left(t\right)=\chi \left(t,{\overset{⌒}{a}}_{y}\left(t\right),u\left(t\right)\right)+K_{3}\left({\bar{a}}_{y}-{\overset{⌒}{a}}_{y}\left(t-\tau_{d}\left(t\right)\right)\right) \end{array},$$

where $\overset{⌒}{x}\left(t\right)$ denotes the predicted value of $x\left(t-\tau_{d}\left(t\right)\right)$, $\overset{⌒}{x}\left(t-\tau_{d}\left(t\right)\right)$ is the time delay signal of $\overset{⌒}{x}\left(t\right)$, and $K={\left[\begin{array}{ccc} K_{1} & 0 & 0\\ 0 & K_{2} & 0\\ 0 & 0 & K_{3} \end{array}\right]}^{T}$ is the predictor gain matrix to be designed, so that $A-KC^{T}$ satisfies the Hurwitz condition.

**Remark** **4.** *The objective of the predictor is that: when*t→∞*,*${\overset{⌒}{x}}_{1}\left(t\right)\to x_{1}\left(t\right)$*,*${\overset{⌒}{x}}_{2}\left(t\right)\to x_{2}\left(t\right)$*and*${\overset{⌒}{x}}_{3}\left(t\right)\to x_{3}\left(t\right)$.

**Remark** **5.** *To achieve the predictor Equation (8), we need to estimate the unknown nonlinear function* χ(x(t),u(t),t) *.*

### 3.2. Adaptive RBF Neural Network Observer

In this section, we use an adaptive RBF neural network method to approximate the unknown nonlinear function χ(x(t),u(t),t). The unknown nonlinear function can be rewritten as follows:

(10) χ(x(t),u(t),t)=f(x(t))+g(x(t))u(t),

where f(x(t)) and g(x(t)) are unknown nonlinear smooth functions; they will be estimated later. We assume no delay in the system; then the expression of the nonlinear system can be rewritten as:

(11) $$\begin{array}{l} \dot{x}\left(t\right)=Ax\left(t\right)+b\left[f\left(x\left(t\right)\right)+g\left(x\left(t\right)\right)u\left(t\right)+d\left(t\right)\right]\\ y\left(t\right)=C^{T}x\left(t\right) \end{array}$$

Then, for Equation (11), an observer is designed as:

(12) $$\begin{array}{l} \dot{\hat{x}}=A\hat{x}+b\left(\hat{f}\left(\hat{x}\right)+\hat{g}\left(\hat{x}\right)u-v\left(t\right)\right)+\Gamma \left(y-C^{T}\hat{x}\right)\\ \hat{y}=C^{T}\hat{x} \end{array}$$

where $\hat{x}$ represents the estimation of the state x, and the functions $\hat{f}\left(\hat{x}\right)$ and $\hat{g}\left(\hat{x}\right)$ are the estimations of unknown nonlinear functions f(x) and g(x), respectively. The robust term v(t), yet to be defined, is a function that provides robustness in the face of bounded disturbances. The observer gain vector Γ is chosen so that the characteristic polynomial of $A-\Gamma C^{T}$ is strictly Hurwitz. The specific expression form of Γ is as follows:

$$\Gamma =\left[\begin{array}{ccc} \Gamma_{1} & 0 & 0\\ 0 & \Gamma_{2} & 0\\ 0 & 0 & \Gamma_{3} \end{array}\right].$$

Defining the state and output estimated errors as $\tilde{x}\left(t\right)=x\left(t\right)-\hat{x}\left(t\right)$ and $\tilde{y}\left(t\right)=y\left(t\right)-\hat{y}\left(t\right)$, yields the error dynamics

(13) $$\begin{array}{l} \dot{\tilde{x}}=\left(A-\Gamma C^{T}\right)\tilde{x}+b\left(\tilde{f}\left(x,\hat{x}\right)+\tilde{g}\left(x,\hat{x}\right)u+d\left(t\right)+v\left(t\right)\right)\\ \tilde{y}=C^{T}\tilde{x} \end{array}$$

where the nonlinear functional estimated errors $\tilde{f}\left(x,\hat{x}\right)$ and $\tilde{g}\left(x,\hat{x}\right)$ are given by

(14) $$\begin{array}{l} \tilde{f}\left(x,\hat{x}\right)=f\left(x\right)-\hat{f}\left(\hat{x}\right)\\ \tilde{g}\left(x,\hat{x}\right)=g\left(x\right)-\hat{g}\left(\hat{x}\right) \end{array}$$

Then the error dynamics may be written as:

(15) $$\tilde{y}\left(s\right)=H\left(s\right)\left(\tilde{f}\left(x,\hat{x}\right)+\tilde{g}\left(x,\hat{x}\right)u+d\left(t\right)+v\left(t\right)\right),$$

where s denotes the differential operator d/dt. The linear transfer function is $H\left(s\right)=C^{T}{\left(sI-A+\Gamma C^{T}\right)}^{-1}b$.

According to the approximation property of an RBF neural network, the continuous nonlinear functions in the observer Equation (12) can be represented by a neural network with constant ideal weights $W^{*}$ and sufficient numbers of basic functions Φ(x), i.e.,

(16) $$\begin{array}{l} f\left(x\left(t\right)\right)=W_{1}^{*}{}^{T}\Phi_{1}\left(x\right)+\varepsilon_{1}\left(x\right),\;\;\;\;\left|\varepsilon_{1}\left(x\right)\right|\leq \varepsilon_{1,N}\\ g\left(x\left(t\right)\right)=W_{2}^{*}{}^{T}\Phi_{2}\left(x\right)+\varepsilon_{2}\left(x\right),\;\;\;\;\left|\varepsilon_{2}\left(x\right)\right|\leq \varepsilon_{2,N} \end{array}$$

where the input vector is $x\in Q_{x}\subset R^{q}$, q is the neural network input dimension, and the neural network approximation errors $\varepsilon_{1}\left(x\right)$ and $\varepsilon_{2}\left(x\right)$ are bounded by known constants $\varepsilon_{1,N}$ and $\varepsilon_{2,N}$. Note that subscripts “1” and “2” indicate quantities associated with f(x) and g(x).

**Assumption** **2** **[43].** *It is assumed that the ideal weights $W_{1}^{*}$ and $W_{2}^{*}$ are bounded by known values, so that*

(17) $${\left\|W^{*}\right\|}_{i,F}\leq W_{i,M},\;\;\;\;i=1,2.$$

Let the neural network functional estimations for f(x) and g(x) be given by

(18) $$\begin{array}{l} \hat{f}\left(\hat{x}\right)={\hat{W}}_{1}{}^{T}\Phi_{1}\left(\hat{x}\right)\\ \hat{g}\left(\hat{x}\right)={\hat{W}}_{2}{}^{T}\Phi_{2}\left(\hat{x}\right) \end{array}$$

where the estimated weights ${\hat{W}}_{1}$ and ${\hat{W}}_{2}$ are provided by the tuning algorithms. Then the expression for the functional estimated errors $\tilde{f}\left(x,\hat{x}\right)$ and $\tilde{g}\left(x,\hat{x}\right)$ defined in Equation (14) are given by

(19) $$\begin{array}{l} \tilde{f}\left(x,\hat{x}\right)=W_{1}^{*}{}^{T}\Phi_{1}\left(x\right)+\varepsilon_{1}\left(x\right)-{\hat{W}}_{1}{}^{T}\Phi_{1}\left(\hat{x}\right)\\ \tilde{g}\left(x,\hat{x}\right)=W_{2}^{*}{}^{T}\Phi_{2}\left(x\right)+\varepsilon_{2}\left(x\right)-{\hat{W}}_{2}{}^{T}\Phi_{2}\left(\hat{x}\right) \end{array}$$

The neural network output errors of the hidden layer are defined as

(20) $${\tilde{\Phi }}_{i}\left(x,\hat{x}\right)=\Phi_{i}\left(x\right)-\Phi_{i}\left(\hat{x}\right),\;\;\;\;i=1,2.$$

Define the weights’ estimated errors as

(21) $${\tilde{W}}_{i}=W_{i}^{*}-{\hat{W}}_{i},\;\;\;\;i=1,2.$$

Adding and subtracting $W_{i}^{*\;\;T}\Phi_{i}\left(\hat{x}\right)$, i=1,2, from Equation (19) yields

(22) $$\begin{array}{l} \tilde{f}\left(x,\hat{x}\right)={\tilde{W}}_{1}{}^{T}\Phi_{1}\left(\hat{x}\right)+W_{1}^{*}{}^{T}{\tilde{\Phi }}_{1}\left(x,\hat{x}\right)+\varepsilon_{1}\left(x\right)\\ \tilde{g}\left(x,\hat{x}\right)={\tilde{W}}_{2}{}^{T}\Phi_{2}\left(\hat{x}\right)+W_{2}^{*}{}^{T}{\tilde{\Phi }}_{2}\left(x,\hat{x}\right)+\varepsilon_{2}\left(x\right) \end{array}$$

Defining the disturbance term $w_{i}\left(t\right)$ as

(23) $$w_{i}\left(t\right)=W_{i}^{*}{}^{T}{\tilde{\Phi }}_{i}\left(x,\hat{x}\right),\;\;\;\;i=1,2$$

and $w_{i}\left(t\right)$ are bounded according to

$$\left\|w_{i}\left(t\right)\right\|\leq \beta_{i},\;\;\;\;i=1,2,$$

with $\beta_{1}\beta_{2}>0$.

Then the proposed observer Equation (12) becomes

(24) $$\begin{array}{l} \dot{\hat{x}}=A\hat{x}+b\left({\hat{W}}_{1}^{T}\Phi_{1}\left(\hat{x}\right)+{\hat{W}}_{2}^{T}\Phi_{2}\left(\hat{x}\right)u-v_{1}-v_{2}\right)+\;\Gamma \left(y-C^{T}\hat{x}\right)\\ \hat{y}=C^{T}\hat{x} \end{array}$$

where $v_{1}$ and $v_{2}$ are robust terms. Then the observation error dynamics become

(25) $$\begin{array}{l} \dot{\tilde{x}}=\left(A-\Gamma C^{T}\right)\tilde{x}+b({\tilde{W}}_{1}{}^{T}\Phi_{1}\left(\hat{x}\right)+w_{1}+\varepsilon_{1}+v_{1}+\left({\tilde{W}}_{2}{}^{T}\Phi_{2}\left(\hat{x}\right)+w_{2}+\varepsilon_{2}\right)u+d+v_{2})\\ \tilde{y}=C^{T}\tilde{x} \end{array}$$

The neural network observer produces a generalized RBF neural network system. Since an RBF neural network is composed of an arbitrarily linear transfer function and a nonlinear mapping operator, the nonlinear plant predefined in Equation (11) can be represented in terms of an RBF neural network.

The output estimated error $\tilde{y}$ is given by

(26) $$\tilde{y}\left(s\right)=H\left(s\right)({\tilde{W}}_{1}{}^{T}\Phi_{1}\left(\hat{x}\right)+w_{1}+\varepsilon_{1}+v_{1}+\left({\tilde{W}}_{2}{}^{T}\Phi_{2}\left(\hat{x}\right)+w_{2}+\varepsilon_{2}\right)u+d+v_{2}),$$

where H(s) is a known proper transfer function with stable poles, and is realized by $\left(A-KC^{T},b,C\right)$. To make the system Equation (26) satisfy the strictly positive real (SPR) condition, we choose a polynomial L(s) so that

(27) $$\tilde{y}\left(s\right)=H\left(s\right)L\left(s\right)({\tilde{W}}_{1}{}^{T}{\bar{\Phi }}_{1}\left(\hat{x}\right)+{\tilde{W}}_{2}{}^{T}{\bar{\Phi }}_{2}\left(\hat{x}\right)u+{\bar{w}}_{1}+{\bar{w}}_{2}u+\;\delta_{1}+{\bar{\varepsilon }}_{1}+\delta_{2}+{\bar{\varepsilon }}_{2}u+{\bar{v}}_{1}+{\bar{v}}_{2}+\bar{d}),$$

where H(s)L(s) is SPR. The “over bar” indicates the signal filtered by $L^{-1}\left(s\right)$ and the terms $\delta_{1}\left(t\right)$ and $\delta_{2}\left(t\right)$ are defined as

(28) $$\begin{array}{l} \delta_{1}\left(t\right)=L^{-1}\left(s\right)\left[{\tilde{W}}_{1}^{T}\Phi_{1}\left(\hat{x}\right)\right]-{\tilde{W}}_{1}^{T}L^{-1}\left(s\right)\left[\Phi_{1}\left(\hat{x}\right)\right]\\ \delta_{2}\left(t\right)=L^{-1}\left(s\right)\left[{\tilde{W}}_{2}^{T}\Phi_{2}\left(\hat{x}\right)u\right]-{\tilde{W}}_{2}^{T}L^{-1}\left(s\right)\left[\Phi_{2}\left(\hat{x}\right)\right]u \end{array}$$

with $\left\|\delta_{i}\right\|\leq c_{i}{\left\|\tilde{W}\right\|}_{F}$, i=1,2.

Then the state-space realization of Equation (27) can be expressed as

(29) $$\begin{array}{l} \dot{\tilde{z}}=A_{c}\tilde{z}+b_{c}({\tilde{W}}_{1}^{T}{\bar{\Phi }}_{1}\left(\hat{x}\right)+{\tilde{W}}_{2}^{T}{\bar{\Phi }}_{2}\left(\hat{x}\right)u+{\bar{w}}_{1}+\;{\bar{w}}_{2}u+\delta_{1}+{\bar{\varepsilon }}_{1}+\delta_{2}+{\bar{\varepsilon }}_{2}u+{\bar{v}}_{1}+{\bar{v}}_{2}+\bar{d})\\ \tilde{y}=C_{c}^{T}\tilde{z} \end{array}$$

where $A_{c}\in R^{n\times n}$, $b_{c}\in R^{n}$, $C_{c}\in R^{n}$ is a minimal state representation of $H\left(s\right)L\left(s\right)=C_{c}^{T}{\left(sI-A_{c}\right)}^{-1}b_{c}$, with $C_{c}={\left[1,0,\ldots ,0\right]}^{T}$.

**Lemma** **1** **[43].** *If a strictly proper rational function $H\left(s\right)=C^{T}{\left(sI-A\right)}^{-1}b$, with A being a Hurwitz matrix, is SPR then there exists a positive-definite symmetric matrix P such that:*

(30) $$A^{T}P+PA=-Q,\;\;Pb=C,$$

*with Q being a positive-definite symmetric matrix.*

**Lemma** **2** **[43].** *Consider the linear time-invariant system in state-space representation:*

(31) $$\dot{x}\left(t\right)=Ax\left(t\right)+Bu\left(t\right),\;\;\;x\left(0\right)=x_{0},$$

*where $x\left(t\right)\in R^{n}$ and $u\left(t\right)\in R^{m}$, and the matrices satisfy $A\in R^{n\times n}$ and $B\in R^{n\times m}$. Then, following [43], every solution x(t) of Equation (31) is such that*

(32) $$\left\|x\left(t\right)\right\|\leq k_{1}+k_{2}{\left\|u\right\|}_{2}^{\alpha },\;\;\;\;\forall t\geq 0,$$

*where $k_{1}$ decays exponentially to zero with $x_{0}$ and $k_{2}$ as positive constants that depend on the eigenvalues of A. Let the robust terms be*

(33) $$v_{i}\left(t\right)=-D_{i}\frac{\tilde{y}}{\left|\tilde{y}\right|},\;\;i=1,2,$$

*where $D_{1}\geq \beta_{1}\sigma_{M}$, $D_{2}\geq \beta_{2}\sigma_{M}u_{d}$, $\sigma_{M}=\sigma_{\max}\left[L^{-1}\left(s\right)\right]$, and $\sigma_{\max}\left[\cdot \right]$ is the maximum singular value. Design the neural network weights adaptive law as*

(34) $$\begin{array}{l} {\dot{\hat{W}}}_{1}=F_{1}{\bar{\Phi }}_{1}\left(\hat{x}\right)\tilde{y}-\kappa_{1}F_{1}\left|\tilde{y}\right|{\hat{W}}_{1}\\ {\dot{\hat{W}}}_{2}=F_{2}{\bar{\Phi }}_{2}\left(\hat{x}\right)\tilde{y}u-\kappa_{2}F_{2}\left|\tilde{y}\right|{\hat{W}}_{2} \end{array}$$

*where $F_{i}=F_{i}^{T}>0$, $\kappa_{i}>0$, i=1,2.*

**Theorem** **1.** *Assuming that the control input* u(t) *is bounded, that is* $\left|u\left(t\right)\right|\leq u_{d}$ *. Consider the nonlinear system Equation (11) and the present adaptive RBF neural network observer system Equation (24), Equation (22) and Equation (34), then the state estimated error* $\tilde{x}\left(t\right)$ *and the neural network weight estimated errors* ${\tilde{W}}_{1}\left(t\right)$ *and* ${\tilde{W}}_{2}\left(t\right)$ *are UUB. See the Appendix B for the proof.*

Therefore, the unknown nonlinear function χ(x(t),u(t),t) can be approximated as:

(35) $$\hat{\chi }\left(x\left(t\right),u\left(t\right),t\right)=({\hat{W}}_{1}^{T}\Phi_{1}\left(\hat{x}\right)+{\hat{W}}_{2}^{T}\Phi_{2}\left(\hat{x}\right)u-v_{1}-v_{2}+\Gamma_{3}\left(y-C^{T}\hat{x}\right)).$$

Based on the approximated nonlinear function, then the proposed predictor Equation (8) can be rewritten in the following forms:

(36) $$\begin{array}{l} \dot{\overset{⌒}{x}}=A\overset{⌒}{x}+b({\hat{W}}_{1}^{T}\Phi_{1}\left(\hat{x}\right)+{\hat{W}}_{2}^{T}\Phi_{2}\left(\hat{x}\right)u-v_{1}-v_{2}+\Gamma_{3}\left(y-C^{T}\hat{x}\right))+K\left(\bar{y}-C^{T}\overset{⌒}{x}\left(t-\tau_{d}\left(t\right)\right)\right)\\ \bar{\overset{⌒}{y}}=C^{T}\overset{⌒}{x}\left(t-\tau_{d}\left(t\right)\right) \end{array}$$

## 4. Simulation

In this section, simulations of two UCAV models with significantly different parameters are taken to verify the effectiveness and universality of the proposed predictor. The main parameters of two UCAVs are listed in Table 1.

**Figure 3:**
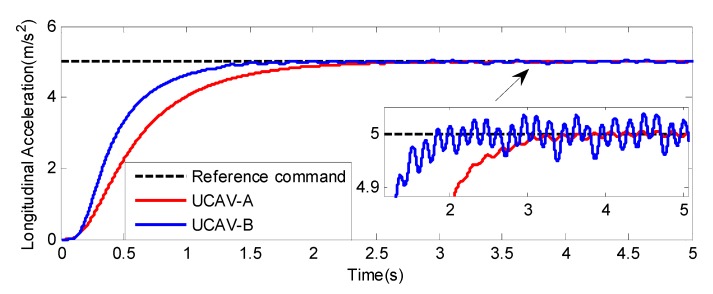
The response characteristics of two UCAVs with control augmentation systems.

The actuators of the two UCAVs are chosen as the same nonlinear model: the backlash is 0.2/57.3 rad and the dead zone is ±0.15/57.3 rad. According to the different dynamic characteristics of the two UCAVs, the appropriate control gains are selected to ensure that the UCAVs’ control augmentation systems have good response characteristics. The response characteristics of two UCAVs with control augmentation systems can be found in Figure 3. We can see that both UCAVs can track the longitudinal acceleration command of $5\;{m/s}^{2}$ stably, but their response characteristics are very different. For UCAV-A, because its flight condition is high altitude and low speed, combined with its dynamic characteristics, the rise time of step response is about 0.9 s. UCAV-B is in the condition of low altitude and high speed, so the control augmentation system has fast response characteristics, about 0.5 s. However, because of its high flight dynamic pressure, UCAV-B is more sensitive to the nonlinearity of the actuator, and the chatter is more obvious in the stable state.

**Figure 4:**
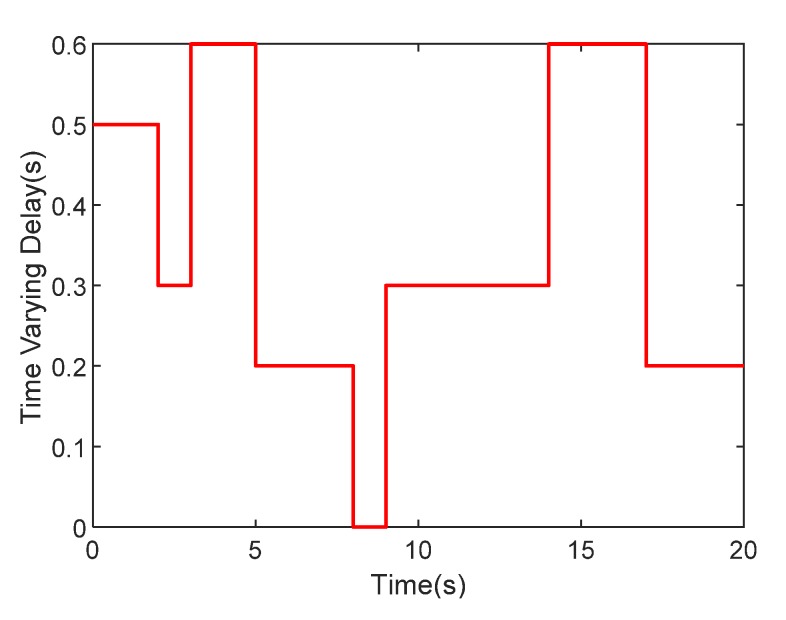
Time-varying delay.

In order to identify the nonlinear models of two UCAVs with control augmentation systems, the RBF neural network adopts the structure of 3-7-1, the input vector has estimated value ${\left[\begin{array}{ccc} {\hat{x}}_{1} & {\hat{x}}_{2} & {\hat{x}}_{3} \end{array}\right]}^{T}$, the initial values of the weights of RBF are set to zero, and the parameters of the Gaussian function are chosen as: the centers are $\eta_{1}=\frac{10000}{3}\left[\begin{array}{ccccccc} -3 & -2 & -1 & 0 & 1 & 2 & 3 \end{array}\right],$ $\eta_{2}=\frac{50}{3}\left[\begin{array}{ccccccc} -3 & -2 & -1 & 0 & 1 & 2 & 3 \end{array}\right],$ $\eta_{3}=\frac{3}{3}\left[\begin{array}{ccccccc} -3 & -2 & -1 & 0 & 1 & 2 & 3 \end{array}\right],$ and the width is $\sigma_{j}=15,\;\;\;\;j=1,\ldots ,7$. We select the linear filter $L^{-1}\left(s\right)=1/\left(s+0.5\right)$, the observer parameters $\Gamma_{1}=40$, $\Gamma_{2}=40$, $\Gamma_{3}=50$, the adaptive parameters $F_{1}=\mathrm{diag}\left[15000\right]$, $F_{2}=\mathrm{diag}\left[15000\right]$, $\kappa_{1}=\kappa_{2}=0.01$ and D=0.5, and the predictor parameters $K_{1}=2$, $K_{2}=1.5$, $K_{3}=1.5$. From the view of engineering, the maximum time delay of the WSN in this piloted mode is about 0.6 s [7], then the time-varying delay $\tau_{d}\left(t\right)$ is chosen randomly in the range [0s,0.6s], which is shown in Figure 4.

**Figure 5:**
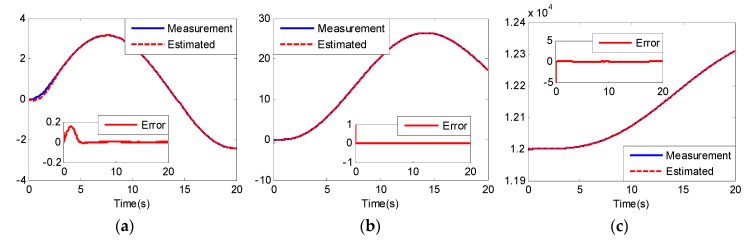
Estimated value $\hat{x}$ and estimated error of UCAV-A: (**a**) longitudinal acceleration $\left({m/s}^{2}\right)$; (**b**) longitudinal velocity (m/s); (**c**) altitude (m).

**Figure 6:**
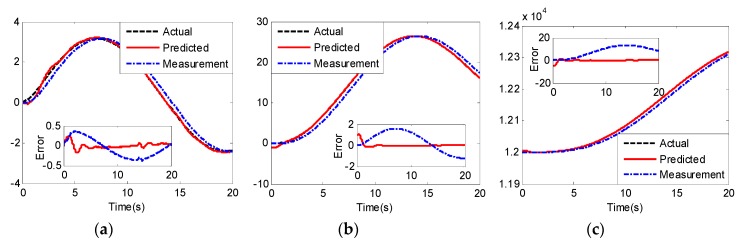
Predicted results $\overset{⌒}{x}$ and predicted error of UCAV-A: (**a**) longitudinal acceleration $\left({m/s}^{2}\right)$; (**b**) longitudinal velocity (m/s); (**c**) altitude.

**Figure 7:**
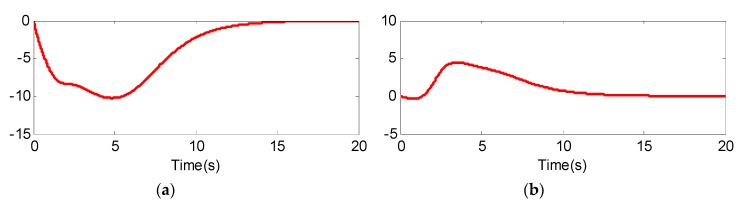
Nonlinear model approximate value of UCAV-A. (**a**) $\hat{f}\left(\hat{x}\right)$; (**b**) $\hat{g}\left(\hat{x}\right)$.

**Figure 8:**
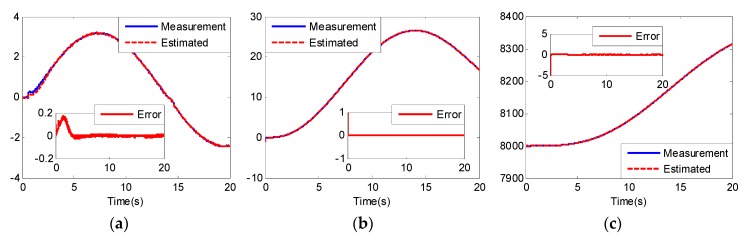
Estimated value $\hat{x}$ and estimated error of UCAV-B: (**a**) longitudinal acceleration $\left({m/s}^{2}\right)$; (**b**) longitudinal velocity (m/s); (**c**) altitude (m).

**Figure 9:**
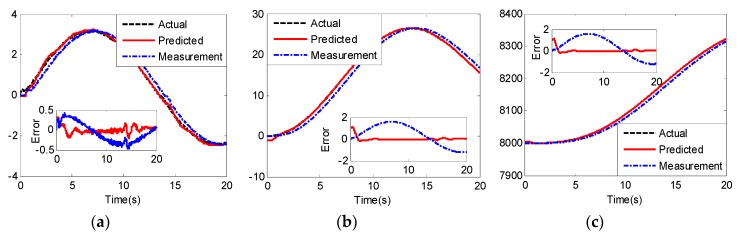
Predicted results $\overset{⌒}{x}$ and predicted error of UCAV-B: (**a**) longitudinal acceleration $\left({m/s}^{2}\right)$; (**b**) longitudinal velocity (m/s); (**c**) altitude (m).

**Figure 10:**
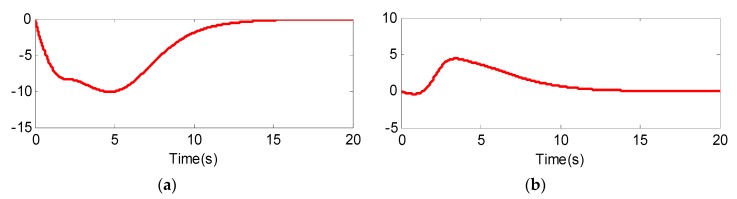
Nonlinear model approximate value of UCAV-B: (**a**) $\hat{f}\left(\hat{x}\right)$; (**b**) $\hat{g}\left(\hat{x}\right)$.

Simulation initial states are set as $x\left(0\right)={\left[\begin{array}{ccc} 8000 & 0 & 0 \end{array}\right]}^{T}$, $\hat{x}\left(0\right)={\left[\begin{array}{ccc} 8001 & 0.5 & 0.1 \end{array}\right]}^{T}$ and $\overset{⌒}{x}\left(0\right)={\left[\begin{array}{ccc} 8001 & 0.5 & 0.1 \end{array}\right]}^{T}$, the remotely piloted command is set as u(t)=3sin(0.25t)+3sin(0.01t), and external disturbance is d(t)=0.1cos(0.7t). The simulation results of UCAV-A and UCAV-B are given in Figure 5, Figure 6 and Figure 7 and Figure 8, Figure 9 and Figure 10, respectively.

From Figure 5 and Figure 8, we can see that although the models of the two UCAVs are quite different, the estimated values can converge to their measured values well with the adaptive RBF neural network observer, and the estimated errors can quickly converge to zero. From Figure 5a and Figure 8a, we can also see that the estimated values of longitudinal acceleration both have the same chatter to the measurement values, and the chatter amplitude of UCAV-B is larger than that of UCAV-A, which is consistent with the analysis result of Figure 3, indicating that RBF has good approximation performance. Figure 7 and Figure 10 show the estimated values of the nonlinear unmodeled dynamics $\hat{f}\left(\hat{x}\right)$ and $\hat{g}\left(\hat{x}\right)$ of two UCAVs; we can see that the estimated values are all smooth and bounded.

The predicted states and predicted errors of UCAV-A are shown in Figure 6. We can see that the predicted values can quickly track the actual values after simulation time 3 s. From the predicted errors and the delay errors compared in Figure 6, we can see that the longitudinal acceleration, longitudinal velocity and altitude delay errors without prediction are $0.4\;m/s^{2}$, 1.8 m/s and 16 m, respectively, and the predictive method makes errors converge to zero. From Figure 9, we can see that the proposed method has the same prediction performance for different UCAVs. In addition, although unmodeled nonlinear dynamics, time-varying delay and external disturbance exist in the simulation, the proposed predictor maintains desirable predictive performance of the actual states.

## 5. Conclusions

This study investigates an adaptive neural network observer-based universal state predictive method for remotely piloted UCAVs, with control augmentation systems in the WSN. The main contributions consist of the following three aspects:

(1) Building a novel universal dynamics model for remotely piloted UCAVs with a control augmentation system. The dynamic process from a pilot longitudinal acceleration command to the UCAV response is modeled as a nonlinear uncertain function, and the longitudinal velocity and altitude are taken as states, which are the first and second integrals of the longitudinal acceleration respectively. This generalized model is suitable for different UCAVs, and does not need to know the model of the UCAV in advance.
(2) An adaptive neural network observer based on Lyapunov is proposed to estimate the nonlinear uncertain model online, and a time-varying state predictor is designed based on the identified nonlinear dynamics model to predict the time delay states. The system stability conditions of the closed-loop system are proven using Lyapunov. The effectiveness and universality is verified by simulation.
(3) The proposed method is widely suited to the application of a remotely piloted vehicle with a control augmentation system in the WSN. Therefore, this method can efficiently resolve the problem of state prediction for the remotely piloted system with time delay.

## Figures and Tables

**Table 1:** The main parameters of two UCAVs.

UCAVs	m(kg)	$I_{yy}\left(\mathrm{kg}\cdot m^{2}\right)$	$\bar{S}\left(m^{2}\right)$	$\bar{c}\left(m\right)$	$H_{0}\left(m\right)$	$V_{0}\left(m/s\right)$
UCAV-A	260	1305	0.124	4.5	12000	246
UCAV-B	480	1680	0.279	6.9	8000	307.5

## Appendix A

The following symbols and abbreviations are used in this manuscript:

$a_{y}$	Longitudinal acceleration
$\bar{c}$	Reference length
$C_{L}\left(\alpha ,\delta_{e},Ma\right)$	Lift coefficient
$C_{M_{yy}}\left(\alpha ,\delta_{e},Ma\right)$	Pitch moment coefficient
g	Acceleration owing to gravity
H	Altitude
$I_{yy}$	Moment of inertia
Ma	Mach
m	Vehicle mass
q	Pitch rate
$\bar{S}$	Reference area
V	Velocity
$V_{y}$	Longitudinal velocity
α	Angle of attack
$\delta_{e}$	Elevator deflection
ϑ	Pitch angle
ρ	Air density
$\tau_{d}$	Time-varying total time delay
$\tau_{down}$	Downlink time delay
$\tau_{up}$	Uplink time delay
Radial-basis-function	RBF
Strictly positive real	SPR
Unmanned aerial vehicle	UAV
Unmanned combat aerial vehicle	UCAV
Uniformly ultimately bounded	UUB
Wireless sensor network	WSN

## Appendix B

**Proof.** Defining the following Lyapunov function

(A1) $$V=\frac{1}{2}{\tilde{z}}^{T}P\tilde{z}+\frac{1}{2}\mathrm{tr}\left({\tilde{W}}_{1}^{T}F_{1}^{-1}{\tilde{W}}_{1}\right)+\frac{1}{2}\mathrm{tr}\left({\tilde{W}}_{2}^{T}F_{2}^{-1}{\tilde{W}}_{2}\right),$$

where $P=P^{T}>0$. Since H(s)L(s) is SPR, there exists $P=P^{T}>0$ such that

(A2) $$A_{c}^{T}P+PA_{c}=-Q,\;\;Pb_{c}=C_{c}$$

for $Q=Q^{T}>0$. Using Equation (A2), the time derivative $\dot{V}$ of V becomes

(A3) $$\begin{array}{l} \dot{V}=\;\frac{1}{2}{\tilde{z}}^{T}\left(A_{c}^{T}P+PA_{c}\right)\tilde{z}+\tilde{y}({\tilde{W}}_{1}^{T}{\bar{\Phi }}_{1}\left(\hat{x}\right)+{\tilde{W}}_{2}^{T}{\bar{\Phi }}_{2}\left(\hat{x}\right)u+{\bar{w}}_{1}+{\bar{w}}_{2}u+\delta_{1}+{\bar{\varepsilon }}_{1}+\delta_{2}+\;{\bar{\varepsilon }}_{2}u+\bar{d}+{\bar{v}}_{1}+{\bar{v}}_{2})+\\ \;\;\;\;\;\;\;\;\;\;\mathrm{tr}\left({\tilde{W}}_{1}^{T}F_{1}^{-1}{\dot{\tilde{W}}}_{1}\right)+\mathrm{tr}\left({\tilde{W}}_{2}^{T}F_{2}^{-1}{\dot{\tilde{W}}}_{2}\right) \end{array}$$

Evaluating Equation (A3) along the trajectories Equation (33) and Equation (34) gives

(A4) $$\begin{array}{l} \dot{V}\leq \;\frac{1}{2}\lambda_{\min}\left(Q\right){\left\|\tilde{z}\right\|}^{2}+\left|\tilde{y}\right|(c_{1}{\left\|{\tilde{W}}_{1}\right\|}_{F}+c_{2}{\left\|{\tilde{W}}_{2}\right\|}_{F}+{\bar{\varepsilon }}_{1}+{\bar{\varepsilon }}_{2}u+\bar{d})+\kappa_{1}F_{1}\left|\tilde{y}\right|\mathrm{tr}\left({\tilde{W}}_{1}^{T}\left(W_{1}^{*}-{\tilde{W}}_{1}\right)\right)+\\ \;\;\;\;\;\;\;\;\;\;\kappa_{2}F_{2}\left|\tilde{y}\right|\mathrm{tr}\left({\tilde{W}}_{2}^{T}\left(W_{2}^{*}-{\tilde{W}}_{2}\right)\right) \end{array}$$

Since

$$-\lambda_{\min}\left(Q\right){\left\|\tilde{z}\right\|}^{2}\leq -\lambda_{\min}\left(Q\right){\left\|\tilde{y}\right\|}^{2},\mathrm{tr}\left({\tilde{W}}^{T}\left(W^{*}-\tilde{W}\right)\right)\leq W_{M}{\left\|\tilde{W}\right\|}_{F}-{\left\|\tilde{W}\right\|}_{F}^{2}$$

we have

(A5) $$\dot{V}\leq \;-\left|\tilde{y}\right|\left(\frac{1}{2}\lambda_{\min}\left(Q\right)\left|\tilde{y}\right|-\sigma_{M}\left(b_{d}+\varepsilon_{1,N}+\varepsilon_{1,N}u_{d}\right)\right)-\left|\tilde{y}\right|\left(\gamma_{1}\left(\alpha_{1}{\left\|{\tilde{W}}_{1}\right\|}_{F}-{\left\|{\tilde{W}}_{1}\right\|}_{F}^{2}\right)-\gamma_{2}\left(\alpha_{2}{\left\|{\tilde{W}}_{2}\right\|}_{F}-{\left\|{\tilde{W}}_{2}\right\|}_{F}^{2}\right)\right),$$

with $\gamma_{i}=\kappa_{i}F_{i}$ and $\alpha_{i}=W_{i,M}+c_{i}/\gamma_{i}$, i=1,2. Squaring the terms ${\left\|{\tilde{W}}_{1}\right\|}_{F}$ and ${\left\|{\tilde{W}}_{2}\right\|}_{F}$, we obtain the following conditions for $\dot{V}$ of V to be negative:

(A6) $$\left|\tilde{y}\right|\geq \max\left\{\frac{4\sigma_{M}\left(b_{d}+\varepsilon_{1,N}\right)+\gamma_{1}\alpha_{1}^{2}}{\lambda_{\min}\left(Q\right)},\frac{4\sigma_{M}\varepsilon_{2,N}u_{d}+\gamma_{2}\alpha_{2}^{2}}{\lambda_{\min}\left(Q\right)}\right\},$$

(A7) $$\begin{array}{l} {\left\|{\tilde{W}}_{1}\right\|}_{F}\geq \frac{1}{2}\alpha_{1}+{\left(\frac{\sigma_{M}\left(b_{d}+\varepsilon_{1,N}\right)}{\gamma_{1}}+\frac{\alpha_{1}^{2}}{4}\right)}^{1/2}\\ {\left\|{\tilde{W}}_{2}\right\|}_{F}\geq \frac{1}{2}\alpha_{2}+{\left(\frac{\sigma_{M}\varepsilon_{2,N}u_{d}}{\gamma_{2}}+\frac{\alpha_{2}^{2}}{4}\right)}^{1/2} \end{array}$$

According to a standard Lyapunov theorem, this demonstrates the UUB of $\left\|\tilde{z}\right\|$, ${\left\|{\tilde{W}}_{1}\right\|}_{F}$ and ${\left\|{\tilde{W}}_{2}\right\|}_{F}$. In order to show the boundedness of the state estimated error $\tilde{x}\left(t\right)$, now analyze the convergence of $\tilde{x}\left(t\right)$. ***step 1:*** solve $\tilde{x}\left(t\right)$ From Equation (24), we can obtain the estimated error as

(A8) $$\dot{\tilde{x}}\left(t\right)=\left(A-\Gamma C^{T}\right)\tilde{x}+b\tilde{u},$$

where $\tilde{u}={\tilde{W}}_{1}^{T}{\hat{h}}_{1}+w_{1}+\varepsilon_{1}+\left({\tilde{W}}_{2}^{T}{\hat{h}}_{2}+w_{2}+\varepsilon_{2}\right)u+d+v_{1}+v_{2}$. When $\tilde{u}$ is not considered, the solution of $\dot{\tilde{x}}=\left(A-\Gamma C^{T}\right)\tilde{x}$ is given by

(A9) $$\tilde{x}\left(t\right)=\tilde{x}\left(0\right)e^{\int_{0}^{t}\left(A-\Gamma C^{T}\right)dt}.$$

Furthermore, the trajectory $\tilde{x}\left(t\right)$ of Equation (A8) can be expressed as

(A10) $$\tilde{x}\left(t\right)=\tilde{x}\left(0\right)e^{\int_{0}^{t}\left(A-\Gamma C^{T}\right)dt}+e^{\int_{0}^{t}\left(A-\Gamma C^{T}\right)dt}\int_{0}^{t}b\tilde{u}\left(\tau \right)e^{-\int_{0}^{\tau }\left(A-\Gamma C^{T}\right)d\tau }d\tau .$$

Let $\Xi \left(t,0\right)=e^{\int_{0}^{t}\left(A-\Gamma C^{T}\right)dt}$, $\Xi \left(t,\tau \right)=e^{\int_{0}^{t}\left(A-\Gamma C^{T}\right)dt-\int_{0}^{\tau }\left(A-\Gamma C^{T}\right)d\tau }$, then the above Equation (A10) can be rewritten as

(A11) $$\dot{\tilde{x}}=\Xi \left(t,0\right)\tilde{x}\left(0\right)+\int_{0}^{t}\Xi \left(t,\tau \right)b\tilde{u}\left(\tau \right)d\tau$$

since

(A12) $$e^{\int_{0}^{t}\left(A-\Gamma C^{T}\right)dt-\int_{0}^{\tau }\left(A-\Gamma C^{T}\right)d\tau }=e^{\left(A-\Gamma C^{T}\right)}=e^{A\left(t-\tau \right)}\times e^{-\Gamma C^{T}\left(t-\tau \right)}\;=m_{0}e^{-\alpha \left(t-\tau \right)},$$

where $m_{0}=e^{A\left(t-\tau \right)}$, $\alpha =\Gamma C^{T}$. Then, $\Xi \left(t,\tau \right)=m_{0}e^{-\alpha \left(t-\tau \right)}$. $m_{0}e^{-\alpha \left(t-\tau \right)}$ is the upper bound of Ξ(t,τ), and $m_{0}$ and α are positive constants. ***step 2:*** convergence of $\tilde{x}\left(t\right)$ Lemma 2 and Equation (A8) yield

(A13) $$\left\|\tilde{x}\left(t\right)\right\|\leq k_{1}+k_{2}{\left\|\tilde{u}\right\|}_{2}^{\alpha },\;\;\;\forall t\geq 0,$$

where

$${\left\|\tilde{u}\right\|}_{2}^{\alpha }=\|{\tilde{W}}_{1}^{T}\Phi_{1}\left(\hat{x}\right)+w_{1}+\varepsilon_{1}+\left({\tilde{W}}_{2}^{T}\Phi_{2}\left(\hat{x}\right)+w_{2}+\varepsilon_{2}\right)u+\;d+{v_{1}+v_{2}\|}_{2}^{\alpha }.$$

Defining $c=w_{1}+\varepsilon_{1}+\left(w_{2}+\varepsilon_{2}\right)u+d+v_{1}+v_{2}$, then

(A14) $${\left\|\tilde{u}\right\|}_{2}^{\alpha }={\left\|{\tilde{W}}_{1}^{T}\Phi_{1}\left(\hat{x}\right)+{\tilde{W}}_{2}^{T}\Phi_{2}\left(\hat{x}\right)u+c\right\|}_{2}^{\alpha }\;\leq {\left\|{\tilde{W}}_{1}^{T}\Phi_{1}\left(\hat{x}\right)\right\|}_{2}^{\alpha }+{\left\|{\tilde{W}}_{2}^{T}\Phi_{2}\left(\hat{x}\right)u\right\|}_{2}^{\alpha }+c_{4}^{\prime },$$

where ${\left\|c\right\|}_{2}^{\alpha }\leq c_{4}^{\prime }$. Since ${\left\|Ax\right\|}_{2}\leq {\left\|A\right\|}_{F}{\left\|x\right\|}_{2}$ and ${\left\|x\right\|}_{2}^{\alpha }=\sqrt{\int_{0}^{t}e^{-\alpha \left(t-\tau \right)}x^{T}\left(\tau \right)x\left(\tau \right)d\tau }$, then

(A15) $${\left\|{\tilde{W}}_{1}^{T}\Phi_{1}\left(\hat{x}\right)\right\|}_{2}^{\alpha }\leq {\left\|{\tilde{W}}_{1}^{T}\right\|}_{F}^{\alpha }{\left\|\Phi_{1}\left(\hat{x}\right)\right\|}_{2}^{\alpha }={\left\|{\tilde{W}}_{1}^{T}\right\|}_{F}^{\alpha }\left\|\Phi_{1}\left(\hat{x}\right)\right\|\frac{1}{\sqrt{\alpha }}\sqrt{1-e^{-\alpha t}}\;\leq {\left\|{\tilde{W}}_{1}^{T}\right\|}_{F}^{\alpha }\frac{1}{\sqrt{\alpha }}c_{5}^{\prime }.$$

Similarly,

(A16) $${\left\|{\tilde{W}}_{2}^{T}\Phi_{2}\left(\hat{x}\right)u\right\|}_{2}^{\alpha }\leq {\left\|{\tilde{W}}_{2}^{T}\right\|}_{F}^{\alpha }\frac{1}{\sqrt{\alpha }}c_{6}^{\prime },$$

where $c_{5}^{\prime }=\left\|\Phi_{1}\left(\hat{x}\right)\right\|\sqrt{1-e^{-\alpha t}}$, $c_{6}^{\prime }=\left\|\Phi_{2}\left(\hat{x}\right)\right\|\sqrt{1-e^{-\alpha t}}u_{d}$. Then

(A17) $${\left\|\tilde{u}\right\|}_{2}^{\alpha }\leq {\left\|{\tilde{W}}_{1}^{T}\right\|}_{F}^{\alpha }\frac{1}{\sqrt{\alpha }}c_{5}^{\prime }+{\left\|{\tilde{W}}_{2}^{T}\right\|}_{F}^{\alpha }\frac{1}{\sqrt{\alpha }}c_{6}^{\prime }+c_{4}^{\prime }.$$

Substituting Equation (A17) into Equation (A13), we get

(A18) $$\left\|\tilde{x}\left(t\right)\right\|\leq k_{1}+k_{2}\left({\left\|{\tilde{W}}_{1}^{T}\right\|}_{F}^{\alpha }\frac{1}{\sqrt{\alpha }}c_{5}^{\prime }+{\left\|{\tilde{W}}_{2}^{T}\right\|}_{F}^{\alpha }\frac{1}{\sqrt{\alpha }}c_{6}^{\prime }+c_{4}^{\prime }\right)\;=c_{3}+\left(c_{4}+c_{5}{\left\|{\tilde{W}}_{1}\right\|}_{F}^{\alpha }+c_{6}{\left\|{\tilde{W}}_{2}\right\|}_{F}^{\alpha }\right)\frac{1}{\sqrt{\alpha }},$$

where $c_{3}=k_{1}$, $c_{4}=k_{2}c_{4}^{\prime }$, $c_{5}=k_{2}c_{5}^{\prime }$, $c_{6}=k_{2}c_{6}^{\prime }$, $c_{4}$, $c_{5}$ and $c_{6}$ are positive constants. From Lemma 2, we can obtain that $c_{3}$ is decaying exponentially to zero owing to the initial condition. The trajectory $\left\|\tilde{x}\left(t\right)\right\|$ is bounded by the weight estimated errors ${\left\|{\tilde{W}}_{1}\right\|}_{F}$ and ${\left\|{\tilde{W}}_{2}\right\|}_{F}$, which are shown to be bounded. □
